# Use of preclinical models for malignant pleural mesothelioma

**DOI:** 10.1136/thoraxjnl-2020-216602

**Published:** 2021-03-10

**Authors:** Marie Shamseddin, Joanna Obacz, Mathew J Garnett, Robert Campbell Rintoul, Hayley Elizabeth Francies, Stefan John Marciniak

**Affiliations:** 1 Wellcome Sanger Institute, Wellcome Genome Campus, Hinxton, Cambridgeshire, UK; 2 Cambridge Institute for Medical Research, University of Cambridge, Cambridge, Cambridgeshire, UK; 3 Department of Oncology, University of Cambridge, Cambridge, Cambridgeshire, UK; 4 Department of Thoracic Oncology, Royal Papworth Hospital NHS Foundation Trust, Cambridge, Cambridgeshire, UK

**Keywords:** pleural disease, mesothelioma, asbestos induced lung disease

## Abstract

Malignant pleural mesothelioma (MPM) is an aggressive cancer most commonly caused by prior exposure to asbestos. Median survival is 12–18 months, since surgery is ineffective and chemotherapy offers minimal benefit. Preclinical models that faithfully recapitulate the genomic and histopathological features of cancer are critical for the development of new treatments. The most commonly used models of MPM are two-dimensional cell lines established from primary tumours or pleural fluid. While these have provided some important insights into MPM biology, these cell models have significant limitations. In order to address some of these limitations, spheroids and microfluidic chips have more recently been used to investigate the role of the three-dimensional environment in MPM. Efforts have also been made to develop animal models of MPM, including asbestos-induced murine tumour models, MPM-prone genetically modified mice and patient-derived xenografts. Here, we discuss the available in vitro and in vivo models of MPM and highlight their strengths and limitations. We discuss how newer technologies, such as the tumour-derived organoids, might allow us to address the limitations of existing models and aid in the identification of effective treatments for this challenging-to-treat disease.

## Introduction

Malignant pleural mesothelioma (MPM) is a relatively rare aggressive cancer.^1^ It is most often caused through inhalation of asbestos fibres, although there is a long latency of >30 years between exposure and the development of disease.^1^ Between 1994 and 2008, 92 253 mesothelioma deaths were reported to WHO, with the majority of cases being from Europe, and age-adjusted mortality rates increased at 5.37% per year.^1^ Following the ban on importation and use of asbestos in many high-income nations, it is predicted that MPM death rates will fall in these nations between 2020 and 2040.^2–5^ For example, in the UK, where the use of all types of asbestos was fully banned in 1999,^6^ the mesothelioma incidence rate is projected to fall by 53% between 2014 and 2035.^7^ Yet, although asbestos use is prohibited in many countries, the industrialisation of low-income andmiddle-income nations is fuelling the use of asbestos and subsequently the incidence of MPM is expected to rise in the coming decades.^8^

MPM affects the mesothelial cells of the pleura and presents as three main histopathological subtypes (table 1): polygonal ‘epithelioid’ MPM is the most common subtype, representing 60% of cases; spindle-shaped ‘sarcomatoid’ MPM accounts for 10% of cases and biphasic mesothelioma, consisting of a mixture of epithelioid and sarcomatoid cells, accounts for the remaining 30%.^9 10^ Mesothelial markers used to identify MPM include calretinin, Wilms’ tumour gene product (WT-1), mesothelin, cytokeratin (CK) 5/6, HBME-1 antigen, thrombomodulin and podoplanin (D2-40).^10^ However, being less differentiated, many sarcomatoid MPM tumours only express CKs and a variable amount of calretinin, but gain expression of vimentin and smooth muscle markers.^10^ The histopathological subtype as well as the stage of disease impacts overall survival with a diagnosis of epithelioid MPM being associated with the longest median survival (13.1 months) and sarcomatoid being associated with the worst survival (4 months).^11^ MPM also has great intratumour heterogeneity, which has been studied using a deconvolution approach which uncovered that all MPM tumours comprised combinations of epithelioid-like and sarcomatoid-like components but the proportions of each are highly associated with prognosis.^12^

**Table 1:** Characteristics of MPM subtypes

Subtype	Morphology	Markers	Prognosis (months)	Genetic alteration*
Epithelioid	Polygonal	Calretinin, WT-1, mesothelin, CK5/6, podoplanin, HBME-1 antigen, thrombomodulin	13.1	*CDKN2A, BAP1, NF2, SETD2, LATS1, LATS2, CDKN2B, MST1, MTOR, STK3, DDX3X, DDX51, SETD5, SF3B1 and TRAF7*
Sarcomatoid	Spindle-shaped	Mostly express only CKs, calretinin, vimentin and smooth muscle	4	*CDKN2A, BAP1, NF2, SETD2, LATS1, LATS2, CDKN2B, MST1, MTOR, SETDB1, TP53, TSC2, ULK2 and SAV1*
Biphasic-E	Mixed	Mixed	8.4	*CDKN2A, BAP1, NF2, SETD2, LATS1, LATS2, CDKN2B, MST1, MTOR, TSC1, STK3, DDX3X, DDX51, SETD5, SF3B1, TRAF7, SETDB1, TP53, ULK2 and SAV1*
Biphasic-S				*CDKN2A, BAP1, NF2, SETD2, LATS1, LATS2, CDKN2B, MST1, MTOR, DDX3X, SETD5, SF3B1, TRAF7, SETDB1, TP53, TSC1, TSC2, ULK2 and SAV1*
Ref	9 10	10	11	34

*Subtypes for genetic alterations refer to the molecular classification based on RNA-sequencing data.

CK, cytokeratin; MPM, malignant pleural mesothelioma; WT-1, Wilms’ tumour.

First-line treatments that have been proposed for MPM include chemotherapy, surgery, radiotherapy, separately or in combination. Surgical procedures such as extrapleural pneumonectomy (EPP) and pleurectomy/decortication have been proposed as curative or palliative approaches, respectively.^13–15^ However, the clinical outcome of the mesothelioma and radical surgery (MARS) trial suggested that EPP within trimodal therapy has no benefit.^16^ The benefit of open pleurectomy/decortication in addition to chemotherapy is currently under investigation in the MARS 2 trial.^17^ The MesoVATS trial previously showed that a video-assisted thoracoscopic partial pleurectomy does not improve overall survival of patients.^18^ At present, the benefits of surgery in MPM remains limited. Radiotherapy primarily has a role in palliation, but has been suggested as an adjuvant to surgery and chemotherapy in multimodality treatment.^13 15^ Combination chemotherapy with pemetrexed and cisplatin has remained unchanged as the standard of care since 2003 following a phase III clinical trial demonstrating superiority of the combination over cisplatin alone (median survival 13.3 months vs 10 months).^19^ In 2016, a phase III trial of the antivascular endothelial growth factor (anti-VEGF) recombinant antibody, bevacizumab, in combination with standard chemotherapy was shown to increase survival in comparison to chemotherapy alone (18.8 months vs 16.1 months) across all MPM subtypes.^20^ This regimen would not be suitable for patients with cardiovascular comorbidities^20^ and currently is not available due to lack of Food and Drug Administration (FDA), European Medicines Agency and National Institute for Health and Care Excellence registration and universal reimbursement.^21 22^ A recent phase lll trial of combined programmed cell death protein 1 (PD-1) inhibition, using nivolumab, and anticytotoxic T-lymphocyte-associated antigen-4 monoclonal antibody ipilimumab, revealed prolonged overall survival compared with the standard chemotherapy regimen.^23^ This drug combination which has been recently approved by FDA^24^ showed more benefit for patients with non-epithelioid tumours,^23^ while non-epithelioid subtypes of MPM were less responsive to chemotherapy or surgery.^25 26^

Comprehensive analysis of MPM samples has revealed the genomic landscape of this disease. A key finding has been that the disease is dominated by loss of function mutations in a number of tumour suppressor genes including: (1) cyclin-dependent kinase inhibitor 2A (*CDKN2A*, alias for *INK4A/ARF*) with homozygous deletion in 45%–49% of cases, encodes two alternative reading frame proteins p14^ARF^ and p16^INK4A^ (involved in the p53 and retinoblastoma protein (RB1) pathways); (2) BRCA associated protein 1 (*BAP1*) mutations are observed in 22%–57% of cases, encodes a tumour suppressor that regulates several processes including the cell cycle, cell death and the response to DNA damage; (3) neurofibromin 2 (*NF2*) is mutated in 19%–50% of cases, encodes Merlin which is involved in the inactivation of the receptor-dependent mitogenic signalling pathway, inhibition of phosphatidylinositol 3-kinase (PI3K) activity and regulation of the hippo pathway.^27–40^ In addition, *TP53* mutations have been reported in 4%–20% of cases.^34 37 41 42^ *CDKN2B* (alias for *INK4B*), which is adjacent to *CDKN2A* and encodes p15^INK4b^ also recurrently shows loss of copy number.^33 34 43^ In the COSMIC database, recurrent mutations have also been found in a further set of genes including *PTEN* and *RB1*.^34^ Comparisons of mutational profiles in the various MPM subtypes reveal common and exclusive mutations. Genetic alterations in *CDKN2A*, *BAP1* and *NF2* are common in all subtypes with higher frequency of *BAP1* and *NF2* in epithelioid and sarcomatoid, respectively.^34^ Other significantly mutated genes include *SETD2, LATS1, LATS2, CDKN2B, MST1* and *MTOR*.^34^ While genetic alterations in *STK3, DDX3X, DDX51, SETD5, SF3B1* and *TRAF7* were exclusively detected in epithelioid and biphasic disease, genetic alterations in *SETDB1, TP53, TSC2, ULK2* and *SAV1* were found only in sarcomatoid and biphasic disease.^34^ Notably, *TP53* mutations were associated with a lower survival rate.^34^ Furthermore, classification of biphasic subtype to epithelioid like (biphasic-E) and sarcomatoid like (biphasic-S) based on RNA-sequencing, found genetic alterations in *STK3* and *DDX51* only in biphasic-E and *TSC2* only in biphasic-S^34^ (table 1).

Conversely, there are few activating oncogenic driver genes or protein-altering mutations in MPM compared with other solid tumours.^34 37^ This unfortunately limits the number of available cancer-selective drug targets since existing drugs mostly target activating oncogenes. Nevertheless, studies of the fundamental biology of MPM have yielded potential novel targeted therapies that are currently being tested in clinical trials, including inhibitors of histone methyltransferase EZH2, focal adhesion kinase, mesothelin, PI3K, mammalian target of rapamycin, PD-1 and programmed death-ligand 1 and anti-angiogenic therapies such as VEGF inhibitors.^44 45^ Unfortunately, many clinical trials in MPM have proved negative.^44 45^ This may reflect the availability of drug resistance pathways to MPM tumours. Furthermore, the lack of biological biomarkers for responsiveness to most targeted therapies has precluded patient stratification and consequently clinical responses restricted to small patient cohorts might conceivably have been missed. However, careful patient selection might improve drug-responsiveness, as suggested by a recent phase IIa trial of a poly-ADP ribose polymerase inhibitor, rucaparib, which demonstrated efficacy in patients with BAP1/BRCA1-deficient malignant mesothelioma.^46^

The development of new therapeutic approaches for MPM requires a larger and more diverse panel of preclinical MPM models to recapitulate the patient population both genomically and histopathologically as well as the ability to model relevant drug response. Consequently, much effort has been invested in establishing two-dimensional (2D) cell lines (cells grown as flat 2D cultures) from primary MPM tumours and pleural effusions.^47 48^ More complex three-dimensional (3D) in vitro models and a number of murine in vivo models have also been developed. Here, we discuss the available in vitro and in vivo preclinical MPM models, highlighting both their strengths and limitations, and the gaps that remain to be filled by improved models.

## Two-dimensional culture of human pleural mesothelioma cells

Many human MPM cell lines have been established from tumour tissue and pleural effusions^48–70^ with success rates ranging from 20% to 84%^54 64 68 70^ (online supplemental table 1). These cell lines represent a spectrum of MPM histopathological subtypes and many harbour the genetic aberrations commonly observed in MPM tumours, including inactivation of *NF2*, *CDKN2A* and *BAP1* genes.^31 39 42 58 71 72^ However, most studies have not compared cell lines with the original tumour from which they were derived to determine how well they recapitulate the genomic and histopathological features. To date, such a comparison has only been conducted once and concordance of genomic alterations was found to be high between the tumour and early passage cell lines, although some single nucleotide variants (SNVs) were found exclusively in the cell lines.^49^ This could represent the expansion of a rare clone present in the tumour or acquisition of new SNVs during in vitro culture. Furthermore, in order to facilitate mesothelioma diagnosis, using a large panel of 61 mesothelioma cell lines from pleural effusions,^73^ a genome-wide analysis has been conducted. As a result, it was shown that the genes *COL3A1*, *SLPI*, *ITLN1* and *CCL2* are expressed preferentially in MPM cells when compared with lung adenocarcinomas.^73^ Accordingly, significantly higher levels of secreted *CCL2* were found in pleural effusions of MPM patients compared with pleural fluid from patients with other metastatic cancers or benign conditions.^73^

10.1136/thoraxjnl-2020-216602.supp1 Supplementary data

The majority of available malignant cell lines can be passaged indefinitely. As such, genomic instability, including polyploidy,^64^ copy number alterations and the emergence of new aberrations^58 74^ have been observed following long-term culture of MPM models. Moreover, kataegis, the accumulation of a large number of single nucleotide substitutions clustered in a single locus was reported in MPM cell cultures but not primary tumour tissue.^49^ It has also been shown recently that newly derived primary mesothelioma cells display a significantly different transcriptome compared with established MPM cell lines.^48^ Furthermore, long-term culturing of primary MPM cells can affect their response to drugs.^75^ Therefore, as cell lines adapt to 2D culture they lose many of the characteristics of the original tumour which can affect their use in research.

It has recently been demonstrated that cell lines represent only part of the subclonal diversity of primary tumours and that different subclones may be dominant in the mesothelioma tumours and derived cell lines.^49^ In line with this, some groups reported the development of two morphologically distant MPM cell lines obtained from the same patient, with differential expression of genes and chromosomal aberrations.^56 65^ Therefore, cell lines fail to recapitulate the complete tumour heterogeneity found in patient tumours.

Early passage, primary MPM cell lines have been exploited in a number of transcriptomic analyses, supporting the identification of putative diagnostic markers and treatment avenues. A recent transcriptomic analysis of a collection of primary MPM cultures, generated in the 1990s from patient samples obtained from French hospitals^76 77^ led to the identification of distinct molecular subgroups of MPM with divergent prognoses. The observed differences could be attributed largely to the varying mutation profiles between subtypes as well as divergent deregulated pathways, including epithelial to mesenchymal transition and transforming growth factor-β signalling.^78^ The molecular subtypes of MPM can also be predicted based on the differential expression of *PPL*, *UPK3B* and *TFP1* genes.^78^ However, none of these studies has been translated to new diagnostic tools nor treatments for MPM.

Early passage MPM cell lines have also been used in drug sensitivity testing in a number of studies,^58 75 79–81^ demonstrating variability in drug response between individuals and indicates the importance of personalised medicine. Integration of drug sensitivity testing of 81 short-term primary mesothelioma cell cultures with gene expression data revealed three response groups corresponding to distinct gene signatures involving the FGF signalling pathway.^79^ High-throughput drug sensitivity testing of a panel of commercial and primary early passage mesothelioma cell lines identified a subgroup of MPM lines highly sensitive to FGFR inhibition as well as death receptor agonist tumour necrosis factor-related apoptosis-inducing ligand (TRAIL), associated with BAP1 loss.^80 81^ A phase Ib clinical trial of a FGF ligand trap in combination with pemetrexed/cisplatin chemotherapy appeared to show durable responses,^82^ but further validation is required before loss of BAP1 can be used as a biomarker for responsiveness to FGF/FGFR inhibitors or TRAIL.

Patient-derived mesothelioma cell lines therefore represent a simple model with which to study MPM biology and sensitivity to therapeutics. However, enthusiasm for their use in guiding personalised medicine must be tempered by important limitations: (i) their proclivity to adapt to 2D culture conditions thereby changing their phenotype, (ii) their failure to recapitulate tumour heterogeneity and (iii) the lack of immune and stromal cell interactions in culture conditions.

## Animal models

Animal models offer the ability to capture some of the complexity of the in vivo tumour environment that is known to contribute to disease progression and drug responsiveness. One major issue of animal models is the time and cost associated with them and subsequently making them unsuitable for large-scale phenotypic screens. Yet, such models are essential for drug testing and have substantially contributed to our understanding of MPM.^83^ Several groups have generated MPM animal models including genetically modified mice, asbestos-induced murine models as well as patient-derived xenografts (PDX) models (table 2).

**Table 2:** List of animal models of MPM

Animal model	Method (if applicable)	Altered genes (if applicable)	Number of MPM patients (if applicable)	Subtypes of MPM (if applicable)	Tumour rate	Median survival (weeks)	Ref
Asbestos/Asbestos--induced	Intrapleural injection of carbon nanotube and asbestos	–	–	NA	9.4%–25%	48–80	98
	Transtracheal intrapulmonary spraying of multiwalled CNT*	–	–	NA	15.8%	70–109	99
Conditional mouse model	Intrathoracic injection with Adeno-Cre virus†	*Nf2;p53* (*hom, hom*)	–	All subtypes	85.5%	19.3	84
		*Nf2;p53* (*het, hom*)	–	All subtypes	61.8%	30.7	
		*Nf2;p53* (*hom, het*)	–	Epithelioid and sarcomatoid	40%	86.4	
		*Nf2;Ink4a/Arf (hom, hom*)	–	All subtypes, predominantly sarcomatoid and biphasic	82.5%	31.4	
		*Nf2;Ink4a/Arf (het, hom*)	–	All subtypes	34.1%	58.6	
		*Nf2;Ink4a/Arf (hom, het*)	–	All subtypes	34.6%	70.7	
		Nf2;p53;Ink4a* (hom, hom, hom)	–	Sarcomatoid and biphasic	100%	11.4	
		Nf2;p53;Ink4a* (hom, hom, het)	–	Sarcomatoid and biphasic	93.8%	NA	
	Intrapleural injection with Adeno-Cre virus	*Nf2;Ink4a/Arf* (*hom, hom*)	–	Predominantly sarcomatoid and some biphasic,^86^ all subtypes^87^	63%^86^	27^86^, 34.8^87^	86 87
		*Bap1;Nf2* (*hom, hom*)	–	Predominantly biphasic and some sarcomatoid	17%	21	86
		*Bap1;Ink4a/Arf* (*hom, hom*)	–	Predominantly sarcomatoid and some biphasic	22%	40	
		*Bap1;Nf2;Ink4a/Arf* (*hom, hom, hom*)	–	Predominantly sarcomatoid and some biphasic,^86^ all subtypes^87^	85%^86^	12^86^, 16.3^87^	86 87
		*NF2; Ink4b/Ink4a/Arf* (*hom, hom*)	–	NA	75%	27.1	87
		*Bap1;NF2;Ink4b/Ink4a/Arf* (*hom, hom, hom*)	–	NA	NA	12.1	
		*Bap1;NF2;Ink4b/Ink4a/Arf* (het, hom, hom)	–	NA	NA	20	
	Intrathoracic injection with Adeno-Cre virus	*Pten;Tp53*	–	Sarcomatoid^88 89^ and biphasic^88^	56%^88^	19.3^88^	88 89
Conditional mouse model exposed to asbestos	Intrapleural injection with Adeno-Cre virus and intrapleural injection of asbestos	*Nf2;p53;Ink4a/Arf*	–	Predominantly epithelioid (>90%) and sarcomatoid	NA	21 (post-Cre-induction without asbestos) and 12 (post-Cre induction with asbestos)	85
Patient-derived xenograft	Subcutaneous	–	50	All subtypes	–	–	105
	Subcutaneous	–	4	Epithelioid	–	–	104

*The numbers indicate the total of pleural and pericardial mesothelioma.

†The numbers indicate thoracic tumours including MPM, rhabdomyosarcomas and schwannomas.

het, heterozygous; hom, homozygous; MPM, malignant pleural mesothelioma; NA, not available.

### Genetically engineered mouse models

MPM mouse models have been established through the alteration of genes known to be involved in human MPM. Mice with mesothelial-specific deletion of *Nf2, p53* and *Ink4a/Arf* were generated using intrathoracic injection of Adeno-Cre virus in homozygous and heterozygous conditional knockout (CKO) mice of *Nf2;p53*, *Nf2;Ink4a/Arf* and *Nf2;p53* carrying an inactive *Ink4a* allele (Nf2;p53;Ink4a*).^84^ Conditional *Nf2;Ink4a/Arf* mice demonstrated increased pleural invasion compared with conditional *Nf2;p53* mice.^84^ Furthermore, homozygous CKO mice Nf2;p53;Ink4a* were highly malignant with invasion in 75% of tumours in both parietal and visceral pleura and had the worst survival with a median survival of around 11 weeks compared with the other homozygous CKO mice (around 19 and 31 weeks in *Nf2;p53*, *Nf2;Ink4a/Arf,* respectively). These indicate that *Ink4a* loss leads to a more aggressive phenotype and poor clinical outcome of MPM.^84^ Immunohistochemistry of the murine mesotheliomas identified the epithelioid MPM phenotype in the CKO mice *Nf2;p53* and *Nf2;Ink4a/Arf* mice, but not in Nf2;p53;Ink4a* mice. Sarcomatoid phenotypes were found in all CKO mice.^84^ In this manner, by inactivating genes known to be mutated frequently in mesothelioma, a powerful model was generated that (i) yielded high incidence of spontaneous mesothelioma development and (ii) gave a short latency for tumour initiation. However, in humans with MPM, it is the epithelioid histological subtype that predominates, while most CKO mice developed sarcomatoid MPM. Only when CKO mice of *Nf2;p53; Ink4a/Arf* were exposed to asbestos using intrapleural injection, the tumours demonstrated predominantly epithelioid subtype.^85^ Furthermore, in contrast to the reported rarity of *p53* mutations in human epithelioid MPM, CKO mice of *Nf2;p53* developed epithelioid MPM. This might reflect species differences with respect to the impact of these genes or relate to different oncogenic mechanisms, such as the prolonged effects of asbestos fibres in human patients versus acute onset of somatic mutations in mice.^84^

Murine models have been generated by CKO in the pleura, of various combinations of the genes most frequently altered in human MPM: *Bap1*, *Ink4a/Arf* and *Nf2*.^86^ Homozygous deletion of each individual gene led to few or no malignant mesotheliomas of the pleura or peritoneum.^86^ Mesotheliomas were observed following homozygous co-deletion of *Bap1;Nf2*, *Bap1;Ink4a/Arf* and *Nf2;Ink4a/Arf* in 17%, 22% and 63% of mice, respectively, rising to 85% of mice in which all three genes were targeted.^86^ The partial penetrance of MPM in all allelic combinations suggests partial redundancy among these tumour suppressors and that additional events are required to drive the phenotype. The median survival of *Nf2;Ink4a/Arf* mice was reproducibly around 27–35 weeks in different studies^84 86 87^ (table 2). The triple CKO mice had a shorter latency (12–16 weeks) compared with double CKO mice (21–40 weeks).^86 87^ Similarly, triple homozygous CKO *Bap1;Nf2;Ink4b/Ink4a/Arf* mice also have a short median survival of 12 weeks and display features similar to those of human MPMs including activation of the PI3K and MAPK pathways, substantial macrophage infiltration and the presence of a significant number of T cells, B cells and natural killer cells.^87^ RNA-sequencing revealed malignant mesotheliomas in the triple CKO mice compared with double CKO of *Nf2;Ink4a/Arf* were enriched in transcripts of genes controlled by polycomb repressive complex 2 (PRC2) including cancer-related genes.^86^ This argues that loss of BAP1, which forms part of a polycomb repressive deubiquitinase complex,^27^ contributes to tumour progression by loss of PRC2-mediated repression of oncogenic genes.^86^ Consequently, these CKO mice are potential models to test therapeutics in these various genetic backgrounds. However, variability in the subtypes of tumours resulting from the same double and triple CKO mice in different studies may complicate interpretation of these results, with non-epithelioid subtypes in one study^86^ and all three histological subtypes in others^84 87^ (table 2).

Given that PI3K signalling pathway appears hyperactivated in many human MPMs, when it should normally be antagonised by PTEN, a mouse model was generated by inactivation of *Pten* and *Tp53* in the mesothelium.^88^ Both peritoneal and pleural malignant mesotheliomas developed in these double KO mice, while *Pten* inactivation alone was not sufficient for tumour development.^88^ Most of the mesotheliomas were non-epithelioid, which is consistent with the low PTEN levels observed in human sarcomatoid tumours and with the association of *Tp53* mutations with non-epithelioid subtypes.^88 89^ This model was associated with MEK/ERK and PI3K activation and, accordingly, inhibition of MEK and PI3K using selumetinib and AZD8186 increased the survival of *Pten;p53* mice.^89^ Although the *Pten;p53* mice provide a relevant preclinical model for mesothelioma with sarcomatoid features, the common somatic genetic alterations found in human MPM are not recapitulated in this model.^88^

### Asbestos/Asbestos-induced murine models

Malignant mesothelioma can also be induced through murine exposure to asbestos fibres. Peritoneal mesothelioma is even rarer than MPM, perhaps because less asbestos reaches the human peritoneum or this might reflect a different mode of pathogenesis entirely. However, most asbestos-induced murine models have been developed by intraperitoneal injection of asbestos and subsequent investigation of extracted mesothelial cells from malignant ascites that formed within the model.^42 90–96^ One reason for this experimental approach is that asbestos exposure by inhalation, which is more physiologically relevant to how MPM develops in humans, results in low pleural tumour burden in murine models.^93^ Several studies demonstrated that inactivation of *Bap1, Nf2, Ink4a/Arf* and *Tp53* led to a higher incidence and rapid progression of malignant mesothelioma in comparison to the wild-type mice treated with asbestos.^42 90–94 97^

Carbon nanotubes (CNT), which share some physical characteristics with asbestos, are increasingly used in medical and commercial applications.^98^ Transtracheal intrapulmonary delivery of multiwalled CNTs in rats induces pleural and pericardial mesothelioma in 16% of rats between 24 and 27 months.^99^ Similarly, in wild-type mice injection of long fibre CNTs into the pleural cavities causes pleural mesothelioma in 10%–25% of animals compared with 9% of mice injected with asbestos.^98^ Progression of murine mesothelioma lesions induced by CNT and asbestos were associated with increased cell proliferation and oxidative damage.^98^ In this model, hypermethylation of the *Ink4a/Arf* locus before tumour development caused loss of p19^Arf^ (homologue to human p14^ARF^) and p16^Ink4a^ protein and led to deletion of *Arf* in end stage mesothelioma, recapitulating an epigenetic feature of human MPM.^98^ The long latency of this model is both its strength and its main weakness. On one hand, it provides a valuable tool to study molecular events that occur during the latency period, such as the hypermethylation causing loss of p19^Arf^, but equally the time required to develop tumours (up to 20 months), make it unsuitable for drug testing. In addition to these issues, the rat model has yet to be characterised extensively at a molecular level.

### Graft models

Subcutaneous or intrathoracic injection of human MPM cell lines into mice are frequently used to study mesothelioma biology and treatment strategies.^100–103^ Highly passaged human mesothelioma cell lines lack many characteristics of their original tumours and, importantly, generate little of the intratumour heterogeneity that is typical of the human disease. To date, there are few studies generating xenografts directly from human MPM tumour tissue or pleural fluid. In one study, subcutaneous xenografts were generated from early passage (below passage 5) primary mesothelioma cultures, derived from the pleural fluid of four patients with MPM and the ascitic fluid of one patient with peritoneal mesothelioma.^104^ A larger bank of xenograft models was established through subcutaneous implantation of fresh tumour material from 50 MPM patients with an engraftment rate of 40% and were subsequently passaged up to five generations.^105^ These xenografts maintained the expression of markers of the primary cells including mesothelin, calretinin, WT-1 and BAP1^104^ and the histological subtype of epithelioid and sarcomatoid in the original tissue.^105^ However, some of biphasic tumours gave rise to xenografts with either epithelioid or sarcomatoid histology only.^105^ While most sarcomatoid and biphasic samples formed xenografts, only one-third of epithelioid samples engrafted successfully and interestingly they were from patients with a poorer outcome.^105^ This limits xenograft models largely to the study of more aggressive forms of MPM. Treatment of a subset of the models across different histological subtypes with cisplatin inhibited growth of 7 of 10 tested PDXs; however, pemetrexed, either alone or in combination with cisplatin, did not affect the growth of these xenografts owing to the metabolism of folate by non-obese diabetic/severe combined immunodeficiency mice, which differs from that in humans.^105^ Furthermore, engrafting the tumour from pleural mesothelium into subcutaneous tissue profoundly changes the tumour microenvironment. Finally, although PDX models are a valuable tool to assess the response of patients to specific treatments, the lack of an immune system limits their utility for testing immunotherapies.

To evaluate immunotherapies for mesothelioma, syngeneic murine models can be used.^106–109^ In these models, murine cell lines are implanted into the immunocompetent host, for example, subcutaneous or intraperitoneal injection of murine mesothelioma cells including: AB1, AB12, AB22 cells in BALB/c mice; AE17 cells in C57BL/6J mice and F4-T2, F5-T1, M5-T1 cells in F344 Fischer rats.^106–114^ Very few studies have used direct implantation of murine mesothelioma lines into the pleural space of mice.^113^ In addition, all these murine mesothelioma cell lines were in fact established from peritoneal mesothelioma following intraperitoneal injection of asbestos.^107 112 115^ While peritoneal and pleural mesothelioma might share some of the same biology, this has yet to be proved. In addition, similar to most available human MPM lines, murine mesothelioma cell lines suffer from having undergone clonal selection leading to adaptation to in vitro culture. Also, as with human cell lines, murine syngeneic tumour models fail to reproduce the intratumour and interpatient heterogeneity seen in the clinic.

## Three-dimensional models

### Spheroids

**Figure 1:**
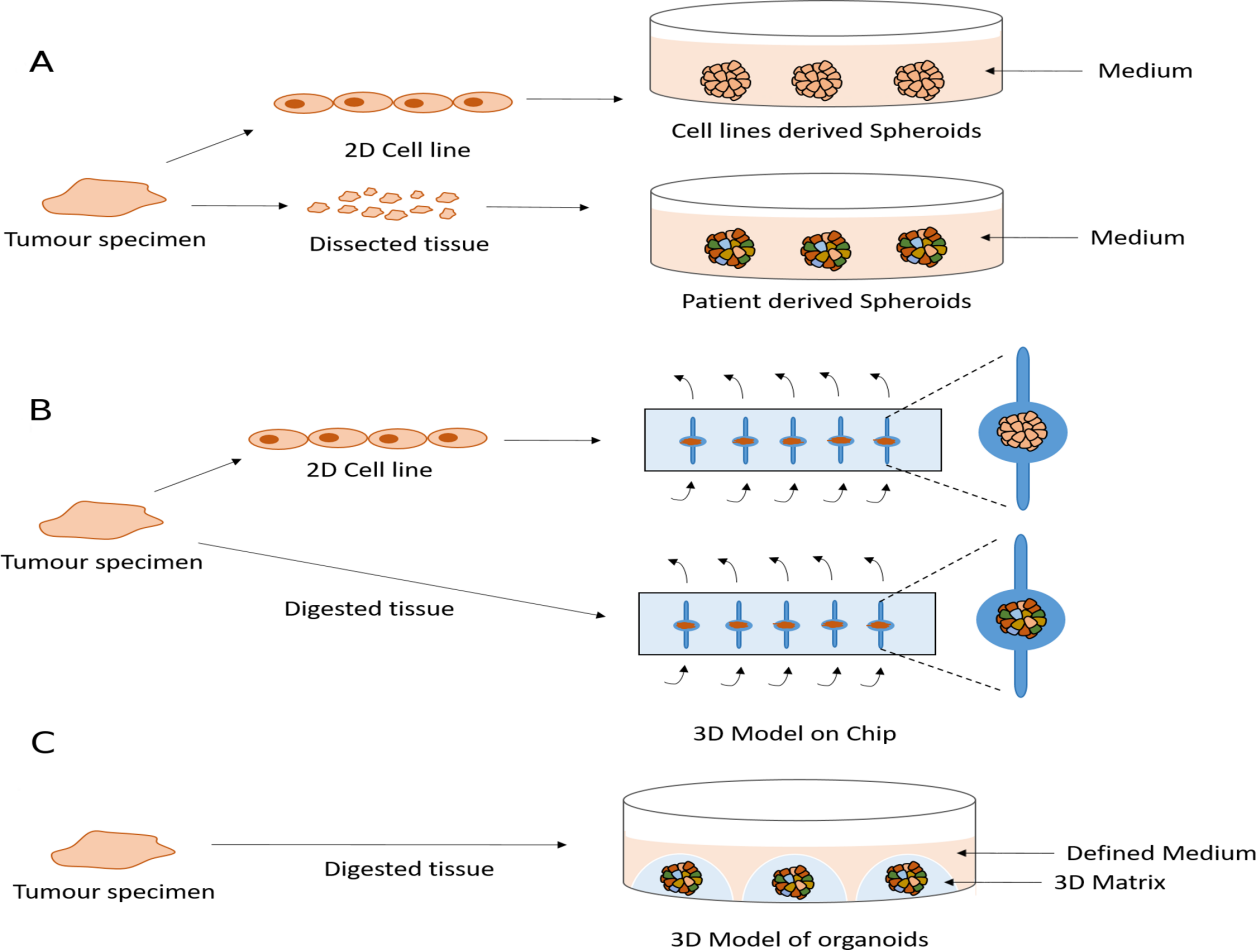
Current and potential future three-dimensional (3D) models of malignant pleural mesothelioma (MPM). (A) Spheroids are obtained by culturing the cell lines or dissected primary tissues as small as 1 mm on a non-adherent or low-adherent plate. (B) Microfluidic chips are implemented to model MPM using cell line-derived spheroids or digested tumour tissue from patients. Arrows shows the medium flow. (C) Potential future 3D model of organoids is obtained using defined medium and a 3D matrix.

Several groups have cultured established mesothelioma cell lines or primary tumour tissue fragments in non-adherent or low-adherent conditions leading to the formation of 3D structures called cancer spheroids. These have been used to study the biology of mesothelioma and test drugs^61 116–119^ (figure 1A). Spheroid cultures recapitulate the resistance of mesothelioma cells to apoptosis more effectively than 2D cultures.^116 120–123^ This may reflect the very different transcriptional profiles that 2D versus spheroid culture conditions generate for the same lines.^124 125^ Indeed, genes involved in apoptosis are downregulated in spheroids.^124^ Although 3D cultures are likely to be an improvement on classical 2D conditions, spheroids derived from cell lines will suffer many of the same limitations as 2D cultures, such as their lack of heterogeneity. By contrast, tumour-derived spheroids may overcome this and be maintained in culture for months. A single report of mesothelioma spheroids grown in vitro describes the retention of a proliferation rate similar to that of the original tumour for 4 weeks.^119^ Unfortunately, there are currently no long-term spheroid models that permit expansion of cell number, which limits the range and number of experiments that can be conducted. Moreover, no studies have yet compared the genomic stability of MPM spheroids and their concordance with the original tumour.

### Tumour on a chip

Microfluidic chips offer another method to model the 3D geometrical shape and the dynamic microenvironment by providing a perfused system mimicking a vascularised tumour.^126^ These devices have been used to develop a 3D model of MPM using either tumour biopsies directly from patients or spheroids derived from cell lines^126 127^ (figure 1B). Although this system has demonstrated potential to predict clinical response to chemotherapy, applicability to long-term culture or indeed systematic drug screening are likely to be challenging.

## Outlook

There has been a disappointing lack of progress in the treatments available for MPM patients and global incidence rates are set to increase. Many clinical trials have been conducted, often based on the results of experiments using simple preclinical models, but they have not translated from model to patient. This suggests that the current preclinical MPM models are not recapitulating human physiology sufficiently well and/or we lack enough models to capture the diversity of the disease. High-quality preclinical models are essential for the development of new treatments for this lethal cancer. Cell lines, spheroids and animal models each have their individual strengths and limitations, so no single model possesses every ideal feature. It is therefore essential that the choice of model is driven by the aim of a given study.

There has been great interest in the recent development of a 3D cell model technology called organoids,^128^ which have overcome many limitations of preclinical models. Organoids are grown in a 3D matrix such as basement membrane extract or Matrigel, enriched for laminins and collagens to resemble the basement membrane^128 129^ (figure 1C). Unlike spheroids, organoids can be propagated for long periods in culture using defined media^128^ making them feasible to biobank and to be an accessible resource. There are now organoid derivation protocols for many epithelial tissues and cancers including pancreas, colon, oesophagus, ovary, breast, prostate, endometrium, liver and lung.^128–137^ These studies have demonstrated how these models can be generated at relatively high success rates (70% to >90%) and importantly recapitulate genomic alterations and histopathological features of the tumour of origin as well as a degree of the subclonal architecture present in the tumour.^128–137^ They are amenable to a number of experimental techniques including medium-throughput drug sensitivity testing^129 138^ and importantly have been shown to recapitulate patient responses to chemotherapy as well as other anticancer agents in the setting of co-clinical trials or a prospective clinical study.^139 140^ Furthermore, methods have been developed that enable the successful co-culture of organoids with other cell types found in the tumour microenvironmant, such as immune and stromal cells, increasing the complexity of organoids culture to better recapitulate the tumour microenvironment.^133 140–144^ Large-scale efforts, such as Human Cancer Models Initiative, are now attempting to derive and characterise panels of cancer organoids that recapitulate the diversity of patient population and to make these models available to the community in order to identify new therapeutic strategies for a variety of cancer types. Unfortunately, to date organoid technology has not been applied to MPM. The development of MPM organoids to provide a strong tool with which to study MPM represents a significant hope for the future.
