# Supplementary material for: Use of preclinical models for malignant pleural mesothelioma
Source: Thorax. 2021 Mar 10;76(11):1154–62. doi: 10.1136/thoraxjnl-2020-216602 (PMC8526879; doi:10.1136/thoraxjnl-2020-216602)
Supplement: Supplementary data [file thoraxjnl-2020-216602supp001.pdf]

Supplementary Table 1. List of human pleural mesothelioma cell lines with characterised histological and genetic background.

Cell line	Origin	Histology/Morphology	Genetic alterations	Ref
MM1	tumour tissue/ pleural effusion	sarcomatoid	loss of *CDKN2A* (7/9); *BAP1* point mutation (4/9); *NF2* loss of function (7/9); kataegis (4/9); chromoplexy (8/9)	[49]
MM2	tumour tissue	desmoplastic		
MM3	tumour tissue	epithelioid		
MM4	tumour tissue	biphasic		
MM5	tumour tissue	biphasic		
MM7	tumour tissue	epithelioid		
MM8	tumour tissue	epithelioid		
MM9	tumour tissue	sarcomatoid		
MM10	tumour tissue	epithelioid		
MPM 1	tumour tissue	epithelioid	NA	[60]
MPM 2	tumour tissue	epithelioid		
MPM 3	tumour tissue	epithelioid		
MPM 4	tumour tissue	biphasic		
MPM 5	tumour tissue	biphasic		
MPM 6	tumour tissue	biphasic		
MPM 7	tumour tissue	sarcomatoid		
MPM 8	tumour tissue	sarcomatoid		
MPM 9	tumour tissue	sarcomatoid		
MESO-3T	tumour tissue	epithelioid	heterozygous loss of *NF2* (5/8), loss of $p16^{INK4a}$ and $p14^{ARF}$ (7/8), structural abnormalities in chr1, 2, 3, 6, 7 and 9	[48]
MESO-7T	tumour tissue	biphasic		
MESO-8T	tumour tissue	epithelioid with sarcomatoid loci		
MESO-9T	tumour tissue	epithelioid		
MESO-12T	tumour tissue	epithelioid		
MESO-14T	tumour tissue	epithelioid		
MESO-17T	tumour tissue	epithelioid		
MESO-27T	tumour tissue	epithelioid		
MM04	pleural biopsy	desmoplastic	chromosomal abnormalities ranging from 41 to 113; monosomy of chr8, 14, 22 (2/5) and 17 (3/5); deletion/translocation in chr1, 5, 6 (epithelioid type) and chr1, 9, 19 (biphasic type); rearrangement of chr1 (5/5) and chr6 (1/5)	[64]
MM05	pleural biopsy	biphasic		
MM12	pleural biopsy	biphasic		
MM13	pleural biopsy	biphasic		
MM1081	pleural biopsy	epithelioid		
PF1038	pleural effusion	unclassified		
HMM-1	pleural effusion	NA	NA	[57]
HMM-3	pleural effusion	NA	NA	
MPM1	pleural effusion	sarcomatoid	hypotetraploid with a modal chr 79	[55]
MPM2	pleural effusion	epithelioid	hypodiploid with a modal chr 40	
MPM3	pleural effusion	biphasic	hyperdiploid with a modal chr 44	
ACC-MESO-1	tumour tissue	epithelioid	*NF2* mutation (1/4); $p16^{INK4a}$ and $p14^{ARF}$ homologous deletion (4/4)	[65,66]
ACC-MESO-4	tumour tissue	epithelioid		

Y-MESO-8	pleural effusion	biphasic		
Y-MESO-9	pleural effusion	NA		
Y-MESO-12	pleural effusion	NA		
Y-MESO-14	pleural effusion	NA		
Y-MESO-21	pleural effusion	NA		
Y-MESO-22	pleural effusion	NA		
Y-MESO-25	pleural effusion	NA		
Y-MESO-26B	pleural effusion	NA		
Y-MESO-27	pleural effusion	NA		
Y-MESO-28	pleural effusion	NA		
Y-MESO-29	pleural effusion	NA		
Y-MESO-30	pleural effusion	NA		
MM-Z03S	pleural effusion	sarcomatoid	both cell lines included chromosomal losses of 1q34~qter, 4, 9p, 10p, 13, 14, 16q, 18, and 22, as well as a complex structural aberration involving chr17. MMZ03E had gains of 3q11q27 and 5p, while MM-Z03S had gain of 9q and losses of 3q27qter, 11q, and 18	[56]
MM-Z03E		epithelioid		
MES-BR 95	pleural effusion	biphasic	NA	[67]
MES-CM 98	pleural effusion	epithelioid		
MES-CG 98	pleural effusion	biphasic		
MES-CE 96	pleural effusion	biphasic		
MES-GF 99	pleural effusion	biphasic		
MES-MM 98	pleural effusion	sarcomatoid		
MES-OC 99	pleural effusion	sarcomatoid		
MES-PP 89	pleural effusion	sarcomatoid		
JL1	tumour biopsy	epithelioid	chromosomal deletions and translocations; chromosomal trisomies and monosomies	[68]
DM3	tumour biopsy	sarcomatoid		
PJ3	pleural effusion	biphasic		
HMMME	pleural effusion	spindle, round and polygonal shape	aneuploidy	[69]
IST-Mes1	pleural effusion	epithelioid-like	monosomy of chr17 (4/4), chr13 (3/4), chr22 (2/4); deletion/translocation of chr6 (4/4), chr1 (3/4), chr1 (3/4)	[70]
IST-Mes2	pleural effusion	epithelioid-like		
IST-Mes3	pleural effusion	spindle-shaped		
MPP89	pleural effusion	epithelioid-like		
H2596	tumour tissue	sarcomatoid	deletion, inversion and translocation affecting chr1, 3, 6, 9; chromosome numbers ranging from 34 to 90; abnormalities in number of chr3, 4, 5, 9, 11, 14, 15, 22	[50]
H2591	tumour tissue	epithelioid		
H2461	tumour tissue	epithelioid		
HP-1	pleural effusion	biphasic		
HP-2	pleural effusion	epithelioid		
H2373	tumour tissue	sarcomatoid		
H2452	tumour tissue	biphasic		
HP-3	pleural effusion	epithelioid		
H2595	tumour tissue	epithelioid		
ZL5	pleural effusion	biphasic	NA	[51]
ZL34	tumour tissue	sarcomatoid		

ZL55	tumour tissue	epithelioid		
ZL92	pleural effusion	not determined		
SPC78	tumour tissue	sarcomatoid		
SPC111	pleural effusion	biphasic		
SPC212	tumour tissue	biphasic		
JU77	pleural effusion	spindle-shaped	deletion of long arm of chr6 (4/5); chromosome 1 rearrangements	[52]
LO68	pleural effusion	spindle-shaped		
ONE58	pleural effusion	spindle-shaped		
NO36	pleural effusion	stellate-shaped		
DeH136	pleural effusion	pleomorphic		
1/M9K	metastasis	biphasic	deletion of long arm of chr5 (5/6); monosomy of chr9 (4/6), chr13 (3/6) and chr22 (2/6); rearrangement of chr1 (3/6)	[53]
2/M10K	metastasis	biphasic		
3/M14K	tumour tissue	epithelioid		
3/M20	pleural effusion	epithelioid		
3/M19	pleural effusion	epithelioid		
4/M33K	tumour tissue	biphasic		
5/M38K	tumour tissue	biphasic		
6/M22K	tumour tissue	biphasic		
6/M19	pleural effusion	biphasic		
Mero-25	pleura autopsy	biphasic	abnormalities in number of ch1, 3, 6, 9, 12 and 22; loss of chr4	[54]
Mero-48a	pleura autopsy	biphasic		
Mero-72	pleura biopsy	biphasic		
Mero-96	pleura biopsy	sarcomatoid		
Mero-14	pleural effusion	NA		
Mero-41	pleural effusion	NA		
Mero-64	pleural effusion	NA		
Mero-82	pleural effusion	NA		
Mero-83	pleural effusion	NA		
Mero-84	pleural effusion	NA		
Mero-95	pleural effusion	NA		
P31	NA	Epithelioid	NA	
VMC6	NA	Epithelioid	NA	[59][61]
VMC11	NA	Biphasic	NA	
VMC12	NA	Epithelioid	NA	
VMC14	NA	Epithelioid	NA	
VMC20	NA	Epithelioid	NA	
VMC23	NA	Epithelioid	NA	
VMC28	NA	Epithelioid	NA	
VMC31	NA	Epithelioid	NA	
VMC33	NA	Epithelioid	NA	
VMC40	NA	Biphasic	NA	
Meso49	NA	Epithelioid	NA	
Meso53	NA	Biphasic	NA	
Meso62	NA	Sarcomatoid	NA	
SPC111	NA	Biphasic	NA	
SPC212	NA	Biphasic	NA	
M38K	NA	Biphasic	NA	
MM288	NA	Epithelioid	NA	[62]

MM317	NA	Epithelioid	NA	
MM404	NA	Epithelioid	NA	
MM473	NA	Epithelioid	NA	
MM487	NA	Biphasic	NA	
MM491	NA	Biphasic	NA	
MM432	NA	Sarcomatoid	NA	
MM472	NA	Sarcomatoid	NA	
H-MESO-1	Tumour	Epithelioid	Pseudotriploid with 68 chromosomes including multiple abnormalities (monosomy, translocations, deletion, trisomy, and tetrasomy)	[63]
